# Editorial and Miscellaneous

**Published:** 1873-12

**Authors:** 


### PART III.

### EDITORIAL AND MISCELLANEOUS.

☞All subscribers and others, will please write their names, P. O., County and State plainly. Remember, this is now required by the Postmaster General.

☞Send five new subscribers and claim the RECORD gratis, or two, and claim Powel's Pocket Formulary.

State when you desire your subscription to commence.

Be sure to give, in your letter, your P. O. to which you wish the journal to go.

All who desire to have a complete volume for 1874, must send in their names by the 15th. Subscription price can be remitted in 30 to 60 days.

☞We can furnish fifty to seventy-five with this volume of the RECORD, '73, either in separate numbers or bound. We would thank our friends to make known this fact. It bound will cost $4.00.

☞We invite our readers and friends everywhere to send us communications. Overhaul your case books and send us your experience.

*To Our Subscribers.*

The fact that but few of our subscribers have signified any intention to cease taking the RECORD at the close of the present volume, and an overwhelming majority of our readers expressing their determination to be subscribers for life, induces us to say that the RECORD will be sent to all of our subscribers as heretofore, with the exception of the few cases who may by letter request us to discontinue. It is therefore understood that every subscriber desires to continue his patronage to the SOUTHERN MEDICAL RECORD who does not specially notify us to the contrary.

*The Closing Year.*

With this number terminates the third year of our journalistic life. Borne along the swift current of time, it becomes us to take a retrospect as, year by year, we leave events behind us, that the trials, disappointments and pleasures of the past may furnish lessons for the future. In looking back, step by step, over our pathway as medical journalists, we lament our short-comings and failures to meet the expectations of so numerous and intelligent a body of medical gentlemen comprising our monthly audience. And yet we feel we have not labored in vain. If we may not at all times, have filled the measure of friendly expectation, we still feel no qualms of conscience, even when we review the unpleasant scenes of a professional war fought and won. The principles upon which, as journalists, we waged battle, have been recognized and adopted. Planting our journal upon these principles, and adhering to truth, pure and simple, we have beheld their triumph, and, under the coercion of truth, their enforcement. No falsehood has ever defaced its pages. No demagoguish swagger, nor disgraceful subterfuges have, at any time, characterized its conduct. We have at no time deceived any one.

With a melancholy heart we pause over the events of the past. We have seen much to sadden, much we would gladly erase from memory's page. Events have come and gone, while the recording angel, whose favor truth alone can propitiate, has placed the never-to-be-effaced seal of approval or condemnation upon. The book is written. Its pages are spotted and marred, or illuminated with beauty and glory by our acts. It is a solemnly serious thing to live! No tears, no anguish of soul can blot one line from the book of our mortal deeds! The good and the true man may—always does—regret that more of good was not the golden fruitage of his every day life; that the path of duty was not more resolutely followed. But he holds within his heart of hearts a conscience void of offence—free from the corruption of an evil intent and of the pollutions of crimes committed. He shall meet no avenger in time, no spoiliation and overthrow from Him who has said, "Vengeance is mine, I will repay." He respires an atmosphere of conscious purity and integrity.

Men, in every sphere of life, may cheat their souls of their birthrights by chosing the evil rather than the good. Medical demagogues may, after a fashion no gentleman could imitate, achieve a name and gather their cohorts, while to the uninitiated they assume virtues which alone crown the brows of the noble and true. They breathe a polluted atmosphere. The deeds of the vile poison the air, carrying moral ruin and death upon all who willingly choose to breathe it. Yet the never erring finger of the faithful one will write their doom, as they have chosen to write their deeds in the every-day acts of life—the vilest of the vile; the chosen disciples of the great deceiver. All earthly aspirations, void of truth and honor, are scorpion stings to the guilty. The wise man has said: "The getting of treasures (or so-called honors) by a *lying tongue* is a vanity tossed to and fro of them that seek *death*." And another has written: "There is no '*peace*' to the wicked."

In every community, the world over, the "tares" are sown with the "wheat." They are known "by their fruits." He who is found wanting where any, the least, principle is involved, is corrupt at heart; destitute of true manhood, and who, oftentimes, will, for a consideration, betray any cause, and disgrace any calling. From "such" all good men should "turn away." Let the honest professional men, the coming year, mark out a new pathway. Let them apply the principles of surgery to remove evils and evil men from societies and from places of position; or, failing, open up a new and better way along which a united brotherhood may walk in harmony and love.

In closing this volume we return our thanks to the many friends who have generously, both by pen and means, sustained the RECORD.

Two of our noble associates have passed away to reap in the spirit land the rewards of good deeds done in tabernacles of clay. A few, also, of our subscribers have departed—passed on, we trust, to the shining shore. Among these was Dr. Harris, of Mississippi, whose wife, in honor to the memory of her husband, still continues the journal he loved so much on earth.

The RECORD has met with many friends. It is established—firmly, we believe—in the affections of the profession. The sweep of its circulation and influence has been onward. It has crossed the border of uncertainty, and now rests "in the valley"—in the land of assured success, the *leading medical journal* of the cotton zone.

For this we thank our friends, one and all. To them is due all its success, and to them it now, in view of the coming year, sends greeting, its warmest thanks, and best wishes for "a happy new year."

*To our Exchanges:*

We thank our exchanges for their kind courtesies of the past year and trust our relations to them and the press generally may be pleasant and satisfactory during the coming year. We wish them all abundant success.

*Our Index.*

We take pleasure in calling attention to the INDEX accompanying this volume. The value of a medical journal is seriously impaired oftentimes by being destitute of a full and well arranged index. We wish to make a bound copy of the RECORD, for easy reference, the equal of any other medical work. Notwithstanding the enormous amount of condensed matter in the RECORD, no reader will be at a loss to find any item in the shortest time. This valuable feature of the RECORD we desire to emphasize. We are endeavoring to give the profession the best and cheapest practical work ever published in this country.

*Dr. J. D. Tucker, of Tennessee,*

Suggests that we furnish specimen copies of the RECORD to all who express a desire to subscribe. It would be impossible to do this without very great pecuniary loss; still we would be willing to do so to a limited extent. The price of a specimen copy is so small (25 cts.) that any one desiring to see it can do so without great loss. We thank Dr. Tucker for his interest in the RECORD, and trust he will send us many subscribers for 1874.

*Thanks to our Associates and Corresponding Editors.*

In closing this volume of the RECORD, we cannot refrain from the agreeable duty of thanking our associate and corresponding Editors for their cordial support during the past year, and their efforts to augment the sphere and increase the usefulness of THE SOUTHERN MEDICAL RECORD.

For the current year, we again solicit their best efforts in our behalf, in furnishing us with appropriate matter for either department of the RECORD. Should they continue to aid us, or will furnish us each with five new names to our subscription list, the RECORD will be sent to them gratis; otherwise they will be expected to contribute the price of subscription. Write us a few articles, or send us five new names and claim the RECORD gratis.

*Additional Editorial Force.*

As the readers of the Record well know, we strive to make it the representative of the medical profession, to make it indispensable to them by its merit alone, and to increase its scope and its practical efficiency by every means in our power. In the exercise of this determination we are happy to announce to our readers a noteworthy accession to our permanent and regular Editorial staff, in the persons of Dr. T. Curtis Smith, of Middleport, Ohio; Dr. Swan M. Burnett, of Knoxville, Tenn.; Dr. A. R. Kilpatrick, of Navasota, Texas; Dr. Alban S. Payne, of Markham, Virginia; Dr. R. C. Word, of Decatur, Ga.; and perhaps a few others.

These gentlemen are distinguished at home and abroad, for eminence in the profession; they are practical scholars, men of enlarged views and great experience, who, through love of the profession, have consented to act as editors for their sections, and will aid us in culling and condensing practical matter and scientific truths, borne monthly upon the bosom of our home and foreign literature. By this invaluable additional "force" the Record will give in every issue a mass of practical information and collection of scientific matter unsurpassed by any medical journal in the United States, and a vast variety of knowledge which intelligent medical men can apply successfully in their daily practice.

*Small-Pox.*

This loathsome disease has made its appearance in several portions of Georgia, especially Macon and Atlanta. In view of its probable spread, every person should be vaccinated, and those who have been, re-vaccinated. Physicians, who are the guardians of the people's health, should see that they obtain pure vaccine virus, and we therefore recommend Dr. E. Cutter & Bro., of Woburn, Mass., to their favorable notice. These gentlemen have fresh and pure virus, from the cow—non-humanized and of undoubted protective value. See advertisement.

*The Meigs County Medical Society.*

The officers of this society, elected at its last annual meeting, are: President, T. Curtis Smith, M. D; Vice President, E. H. Trickle, M. D.; Secretary, C. H. Tidd, M. D.; Treasurer, W. B. Guthern, M. D.

This society is in a healthy and prosperous condition, with a large roll of members, and from what we can learn, is the most active and best attended society of the kind in South Ohio.

*An Honor Conferred.*

The Editors of the RECORD have been elected Corresponding members of the MEIGS AND MASON ACADEMY OF MEDICINE, Middleport, Ohio. They desire to make acknowledgment of the distinguished honor conferred, and shall ever remember the fact, and hold in esteem the medical brethren with whom they have thus been brought into communion. The Society and its members have our heartfelt thanks and best wishes for its success — a success which, if as brilliant in the future as in the past, is destined to make it the most useful and distinguished of all our local societies.

*Communications,*

Have been received from the following gentlemen, to whom the editors return their sincerest thanks:

Ophthalmia Neonatoram, by J. C. Bishop, M. D., Ohio; Infant Management, by T. K. Bailey, M. D., Tenn.; Extracts, by Swann M. Barnett, M. D., Tenn.; Case of Cancer of the Pancreas and Modified Transfusion of Blood, by Ephriam Cutter, M. D., Mass.; Diphtheria, by L. B. Bouchelle, M. D.; Chloral in Cholera Morbus, by L. B. Bouchelle, M. D., of Georgia; Ossification of placenta, by A. S. Smythe, M. D., of Miss.; Fluid Extract of Phytolacca Decandra, by Geo. A. Dyer, of Ind.; Position in Labor, by W. B. Williams, M. D., of Miss.; Hydrophosphite of Soda and Quinine in Enlargement of Spleen, by R. S. Hallsey, M. D., of N. C.; A Woman with Three Breasts, by A. R. Kilpatrick, M. D., of Texas; Correspondence by Fed. Horner, Jr., of Va.; Correspondence by B. P. Reese, M. D., of Va.; Belladonna in aphonia cases, by T. Curtis Smith, M. D., of Ohio; In-growing Toe-Nail, by J. Q. A. Hudson, M. D., of Ohio; Gelseminum, by G. D. Hodge, M. D., of Arkansas.

*Hearth and Home.*

We commend this beautiful journal to our readers as one of the ablest, purest, and most carefully edited of the literary and home weeklies. Its stories are not of the trashy, sensational character, yet are of absorbing interest, and will be read by every member of the family with pleasure and profit. Its editorials are able, timely, and independent. The best American and foreign writers contribute to its columns; and, take it all in all, it is as near a perfect Home Paper as is published in this or any other country. No family should be without it. ORANGE JUDD COMPANY, Publishers, 245 Broadway, New York.

*Our Contributors.*

We desire to express our gratitude to the large number of gentlemen, named below, who have generously given their support to the RECORD by contributing articles for its pages during the year 1873. They have added much to the interest, value and reputation of our journal, and we cordially invite them (and all others) to aid us (and thereby benefit the profession) during the coming year:

Jas. T. Allford, M.D., L. B. Anderson, M.D., Robert Battey, M. D., Louis B. Bouchelle, M.D., Swann M. Burnett, M. D., A. W. Calhoun, M.D., T. A. Cooke, M.D., B. I. A. Cull, M.D., L. H. Desseau, Ely Van De Warker, M.D., A. Donald, M.D., W. T. Grant, M.D., W. W. Harrison, M.D., T. J. Heard, M.D., E. T. Henry, M.D., A. S. Helmick, M.D., S. V. D. Hill, M.D., Fred. Horner, Jr., M.D., G. D. Hodge, M.D., A. R. Kilpatrick, M.D., J. G. Knox, M.D., W. L. Lipscombe, M.D., John E. Lochridge, M.D., Charles S. Lewis, M.D., Thos. S. Mitchell, M.D., A. Murray, M.D., A. S. Payne, M.D., Benj. Rhett, M.D., S. S. Satchwell, M.D., J. W. M. Shattuck, M.D., T. Curtis Smith, M.D., A. G. Smythe, M.D., A. J. Steele, M.D., W. T. Taylor, M.D., C. H. Tidd, M. D., J. D. Tucker, M.D., James Tyson, M.D., E. M. Vasser, M.D., W. R. Whitehead, M.D., J. H. Wier, M.D.

*Scribner's Monthly*

Is to be made more attractive than ever during 1874. A charming serial story by Miss Trafton, entitled "Katherine Earle," with brilliant novelettes and short stories by Saxe Holm, Bret Hart, and other delightful story-tellers, will be attractions. Benj. F. Taylor will give a series of poems, with illustrations—"Old Time Music." "The Great South," the most important and expensive series of illustrated papers ever undertaken by any magazine, will be continued through the year. Scribner's is peerless, and should find a welcome in every home. Its purity of language and fidelity to christian truths, forms a delightful feature of its editorial management. Physicians should remember that Dr. Holland is one of them—an M. D., and with the RECORD subscribe for Scribner. Price $4.00. Address Scribner & Co., 654 Broadway, N. Y.

*Vick's Floral Guide for 1874.*

This beautiful quarterly issued by the great seedsman, Jas. Vick, of Rochester, N. Y., is on our table. The number before us contains 200 pages, with 500 engravings and colored plates. Price 25c. a year.

*Obituary.*

Died August 12th, 1873, in the 56th year of his age, one of our associates, Dr. Charles S. Lewis, of Brunswick Co., Va., after a long illness. He was the eldest son of Dr. Henry Lewis, of Lawrenceville, Va. Dr. Lewis was one of the earliest and best friends of the writer, who can bear witness to the great moral worth, high ability, and excellent intellectual qualities of the deceased. As a medical man he had perhaps no superior in his native State; as a friend and citizen no man more fully than he set an example of excellence; in manners he was modest and unobtrusive, ever true to the dictates of truth, unswerving in his fidelity to the principles of right; honored by all who knew him, and in all respects a perfect gentleman. He died a Christian, and in the full hope of a blessed immortality. To his affectionate wife and dear children, his venerable father and estimable brothers, we extend our sincerest condolence, with the hope that their grief will be assuaged in contemplating the ineffable bliss now enjoyed by their beloved one in the realm of Heaven, where his happy spirit, awaiting their coming, rests under the shadow of the Tree of Life in glory and in peace forever.

